# Laparoscopy as a Diagnostic and Definitive Therapeutic Tool in Cases of Inflamed Simple Lymphatic Cysts of the Mesentery

**DOI:** 10.1155/2015/325939

**Published:** 2015-05-06

**Authors:** Abdelrahman Abdelaal, Ibnouf Sulieman, Zia Aftab, Ayman Ahmed, Saif Al-Mudares, Mohannad Al Tarakji, Ahmad Almuzrakchi, Adriana Toro, Isidoro Di Carlo

**Affiliations:** ^1^Department of General Surgery, Hamad General Hospital, Doha, Qatar; ^2^Department of Radiology, Hamad General Hospital, Doha, Qatar; ^3^Department of Surgery, Taormina Hospital, 98034 Taormina, Italy; ^4^Department of Surgical Sciences, O.T. and A.T., University of Catania, 95126 Catania, Italy

## Abstract

Mesenteric cysts are rare benign abdominal tumors. These cysts, especially those of lymphatic origin, very rarely become inflamed. The diagnosis of inflamed lymphatic cysts of the mesentery may be difficult. We herein report two cases of inflamed simple lymphatic cysts of the mesentery definitively diagnosed and excised by laparoscopy.

## 1. Background

Mesenteric cysts are rare benign abdominal tumors with an incidence of 1 case in 27,000 to 250,000 hospital admissions [1, 2]. Their origin remains controversial. Some authors consider that mesenteric, omental, and retroperitoneal cysts have the same origin [1, 3]. Other authors define these various mesenteric cysts as separate entities [4]. Mesenteric cysts are divided into two main categories: those of lymphatic origin and those of mesenteric origin. They are usually located in the small bowel mesentery or right mesocolon. Mesenteric cysts, especially those of lymphatic origin, rarely become inflamed. In such cases, diagnosis may become difficult. We herein report two cases of inflamed simple lymphatic cysts of the mesentery definitively diagnosed and excised by laparoscopy.

## 2. Case 1

A 28-year-old woman presented to the emergency room with left upper quadrant pain that radiated to the epigastrium. The pain increased with side-to-side movement and was alleviated by lying down. The patient had no history of nausea, vomiting, or recent changes in bowel habits. She had experienced no similar attacks in the past but had had a spontaneous abortion 2 weeks prior to presentation secondary to a bicornuate uterus anomaly.

**Figure 1:**
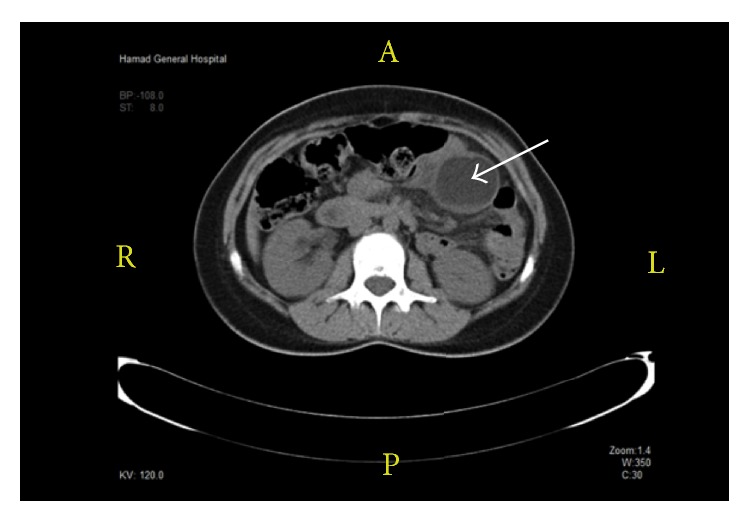
Case 1. Computed tomography scan showing mesenteric cyst (white arrow).

No other abnormalities were found on physical examination at the time of admission. Laboratory studies showed mild leukocytosis (12,000/*μ*L) with normal liver function test results and normal amylase and lipase levels. An emergency contrast-enhanced computed tomography scan of the abdomen showed a fairly well-circumscribed, 4.1–5.0 cm, predominantly fat-containing structure in the left upper abdomen, close to the small bowel loops and mesentery. The lesion was devoid of calcification and demonstrated subtle peripheral enhancement and a small layer of dependent debris (Figure 1). It was diagnosed as a mesenteric cyst.

**Figure 2:**
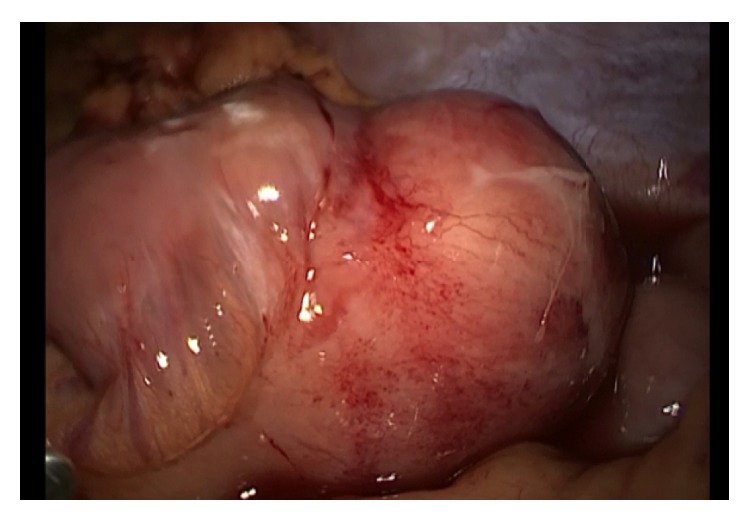
Intraoperative view of mesenteric cyst.

**Figure 3:**
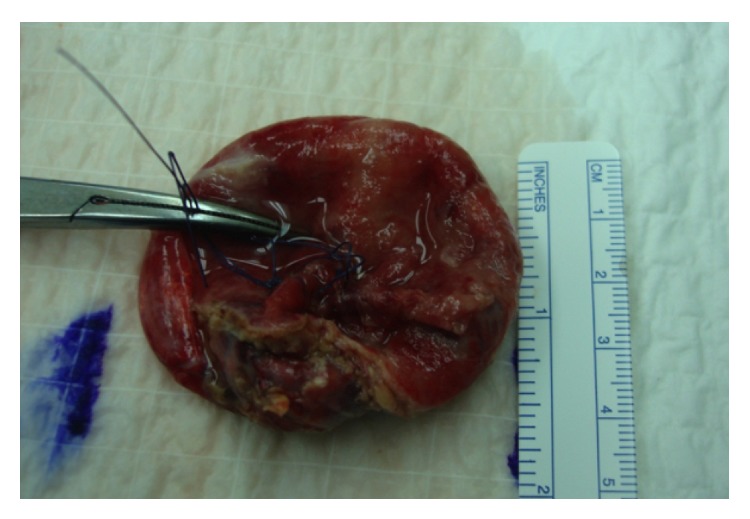
Gross specimen.

The patient was hospitalized for laparoscopic exploration and excision of the nonperforated mesenteric cyst (Figures 2 and 3). Histopathologically, the lesion exhibited a benign fibrous wall with diffuse surface necrosis and fibrin deposition. Evidence of acute inflammation was present, and this inflammation was surrounded by mesenteric fatty tissue, which was consistent with a simple lymphatic cyst of the mesentery. The postoperative course was uneventful. The patient was discharged on postoperative day 3 and followed up later in the outpatient clinic with no infectious complications.

## 3. Case 2

A 24-year-old previously healthy man with no medical or surgical diseases presented to the emergency room with a 2-day history of severe abdominal pain located in the epigastrium and left upper quadrant. The pain radiated to the left shoulder. It was associated with nausea and vomiting, but no change in bowel habits. He was a chronic smoker.

**Figure 4:**
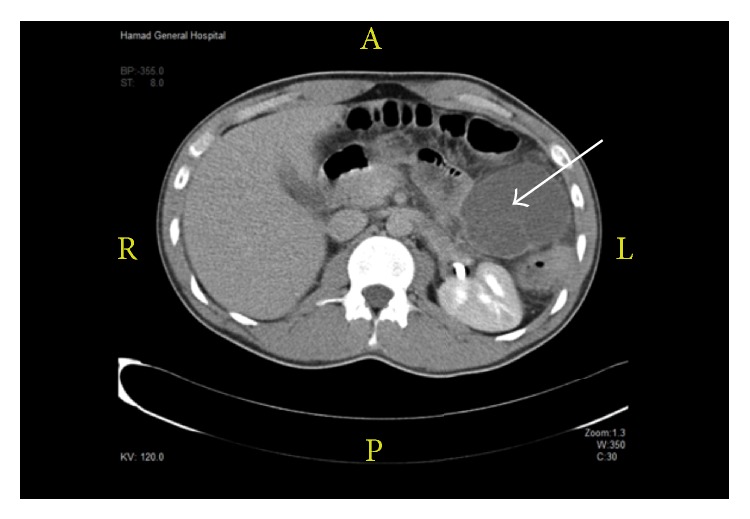
Case 2. Computed tomography scan showing mesenteric cyst (white arrow).

On admission, the patient was febrile (39.2°C) and tachycardic (109 bpm). Physical examination revealed left upper quadrant tenderness, but no palpable mass; bowel sounds were heard. The only significant laboratory finding was leukocytosis (17,200/*μ*L). Abdominal contrast-enhanced computed tomography showed a cystic lesion measuring 7.2 × 6.7 cm adjacent to the splenic flexure and extending to the pancreatic tail region. Minimal surrounding fat stranding was noted (Figure 4).

**Figure 5:**
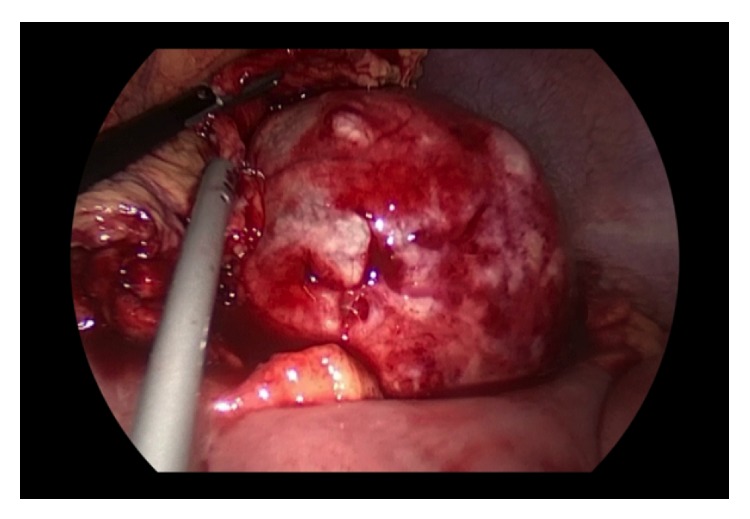
Intraoperative view showing intense inflammation of the mesenteric cyst.

The patient's lesion was diagnosed as a mesenteric cyst, and he underwent laparoscopic exploration and excision of the nonperforated cyst (Figure 5). Histopathological examination of the cyst wall revealed areas of hemorrhage, necrosis, fibro-adipose tissue, and marked acute inflammation. The definitive diagnosis was an inflamed simple lymphatic cyst of the mesentery. The postoperative course was uneventful without any infectious complication, and the patient was discharged on postoperative day. He was followed up 2 weeks later in the outpatient clinic.

## 4. Discussion

Mesenteric cysts were first reported in 1507 by the Italian anatomist Benevieni [5]. The first surgical excision was performed by Talliaux in 1880 [6]; since then, almost 900 cases have been reported.

Mesenteric cysts mainly occur in the fifth decade of life and predominantly in women. However, they may also occur in children. The pathogenesis of mesenteric cysts is ambiguous and has been presumed to involve a congenital abnormality in the development of the lymphatics, resulting in congenitally malformed, misplaced, or malpositioned lymphatic tissue [7].

According to their etiology characteristics mesenteric cysts are classified as (1) fetal and developmental cysts, (2) traumatic or acquired cysts, (3) neoplastic cysts, and (4) infectious or degenerative cysts [8]. But according to their histopathological features the mesenteric cysts may have the following classification: cysts of lymphatic origin (simple lymphatic cyst and lymphangioma), cysts of mesothelial origin (simple mesothelial cyst, benign cystic mesothelioma, and malignant cystic mesothelioma), cysts of enteric origin (enteric cyst and enteric duplication cyst), cysts of urogenital origin, mature cystic teratoma (dermoid cyst), and pseudocysts (infectious and traumatic cysts) [9]. However, a more complete classification was recently published [4, 10, 11], and we have modified this classification with the addition of another type (Table 1).

Mesenteric cysts are usually asymptomatic. When symptoms are present, they are unspecific in 40% to 45% of patients (e.g., nausea, vomiting, or abdominal discomfort, pain, or distension) [12]. Affected patients may present with acute conditions such as inflammation or abscess formation or rupture. Simple lymphatic cysts rarely became inflamed. However, inflammation developed in both of our patients. Cysts can be infected and this mechanism is currently unexplained. In the case of lymphangiomas (the most common mesenteric cysts) some patent lymphatic channels can be extended from the cyst to the small bowel wall; the bacteria from the intestine can migrate through the lymphatic causing lymphangitis and consequently the infection of the cysts. A second cause of infection is the puncture both for diagnosis and for evacuation. Finally cyst treated with marsupialization can recur with infection [13]. Both last procedures can be utilized preoperatively in case of simultaneous small bowel resection, but infectious complications can occur and for this reason are strongly discouraged [12].

Laboratory tests frequently do not supply any specific information. As in our patients, however, inflammation may be present as evidenced by leukocytosis. Ultrasonography and computed tomography can be used as diagnostic tools. They can show variable features suggestive of fluid content, a surrounding capsule with smooth margins, and endovascular abnormalities; however, they can also result in a missed diagnosis. Magnetic resonance imaging can be used as another diagnostic modality [14]. Such diagnostic tools can help to determine the size of the mesenteric cysts. Simple lymphatic cysts are usually 1 to 5 cm in diameter. Larger cysts are usually multiloculated lymphangiomas or benign cystic mesotheliomas. One of our patients had a cyst of 7 cm in diameter, and this was one of the reasons why the diagnosis was not achieved preoperatively but instead with pathological examination.

One of the mesenteric cysts in the present report was located in the small bowel mesentery, the most common site for such cysts. The second cyst was located in the transverse mesocolon, a less common site. The most common sites for mesenteric cysts are the small bowel mesentery, mesocolon, and rectum [12].

The standard treatment of mesenteric cysts is surgical resection, which is mandatory in patients with large cysts to confirm the benign nature of the disease and exclude any malignancies. Indeed, complete resection of the cyst is required to avoid recurrence, especially in cases of malignancy [1].

Laparotomy has also been a treatment choice for many years. After the first report of laparotomy in 1993 by Mackenzie [11], the laparoscopic approach became increasingly more popular. The preferred method of excision is enucleation, which is usually easily performed; however, in cases involving firm adhesion because of inflammation or in cases of malignancy, the procedure can become difficult. If extreme difficulties are encountered, conversion to an open procedure must be quickly adopted. Laparoscopic treatment was performed in both of our cases, even in the presence of surrounding inflammation.

## Figures and Tables

**Table 1:** Classification of mesenteric cysts and cystic tumors.

Cysts of lymphatic origin	Simple lymphatic cists
	Lymphangioma

Cysts of mesothelial origin	Simple mesothelial cyst
	Benign cystic mesothelioma
	Malignant cystic mesothelioma

Cysts of enteric origin	Enteric duplication cyst
	Enteric cyst

Mucinous cystic neoplasms	Mucinous cystadenoma
	Borderline malignant mucinous cystic neoplasm
	Mucinous cystadenocarcinoma

Cysts of urogenital origin	

Miscellaneous neoplasms	Mature cystic teratoma (dermoid cysts)
	Neuroendocrine carcinoma
	Cystic spindle cell tumor

Nonpancreatic pseudocysts	Cysts of traumatic origin
	Cysts of infectious origin

Nonneoplastic cysts	Hydatid cyst
	Mycotic cyst
	Parasitic cyst
	Tuberculous cyst
	Cystic degeneration of lymph nodes and other tissues
